# Fidelity or love the one you're with? Biotic complexity and tradeoffs can drive strategy and specificity in beetle‐fungus by‐product mutualisms

**DOI:** 10.1002/ece3.10345

**Published:** 2023-07-22

**Authors:** Diana L. Six, Peter H. W. Biedermann

**Affiliations:** ^1^ Department of Ecosystem and Conservation Science University of Montana Missoula Montana USA; ^2^ Chair of Forest Entomology and Protection University of Freiburg Freiburg Germany

**Keywords:** ambrosia beetle, bark beetle, by‐product mutualism, Scolytinae, symbiosis, tradeoffs

## Abstract

By‐product mutualisms are ubiquitous yet seldom considered in models of mutualism. Most models represent conditional mutualisms that shift between mutualism and antagonism in response to shifts in costs and benefits resulting from changes in environmental quality. However, in by‐product mutualisms, benefits arise as a part of normal life processes that may be costly to produce but incur little‐to‐no additional costs in response to the interaction. Without costs associated with the interaction, they do not have antagonistic alternate states. Here, we present a conceptual model that differs from traditional conditional models in three ways: (1) partners exchange by‐product benefits, (2) interactions do not have alternate antagonistic states, and (3) tradeoffs are allowed among factors that influence environmental quality (rather than all factors that contribute to environmental quality being combined into a single gradient ranging from high to low). We applied this model to bark and ambrosia beetles (Curculionidae: Scolytinae), a diverse group that associates with fungi and that has repeatedly developed two distinct pathways to by‐product mutualism. We used independent axes for each major factor influencing environmental quality in these systems, including those that exhibit tradeoffs (tree defense and nutritional quality). For these symbioses, tradeoffs in these two factors are key to which mutualism pathway is taken.

## Graphical abstract

In by‐product mutualisms, benefits arise as a part of normal life processes that do not incur additional costs. They are not conditional where outcomes shift between mutualism and antagonism as environmental context varies. Most models are conditional and a poor fit for by‐product mutualisms. Here, we build on a prior model, removing conditionality and incorporating tradeoffs in environmental quality to support testing hypotheses on by‐product mutualism development and partner fidelity.

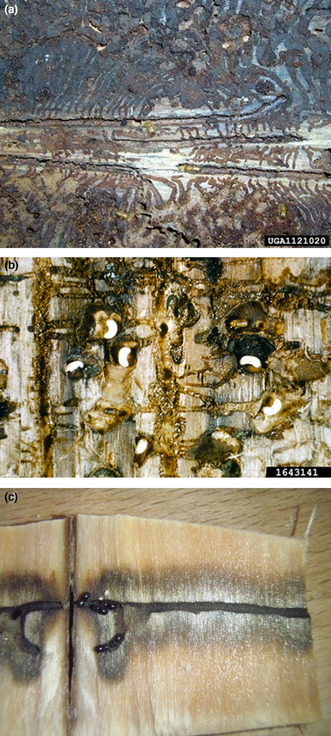

## 1 INTRODUCTION

Mutualistic partnerships have opened or expanded niches for many hosts and their symbionts and support much of Earth's biodiversity (Borges, 2017; Weiblen & Treiber, 2015). While the importance of mutualisms is now well recognized, the ecological and evolutionary processes that have led to their formation and maintenance (Bourke, 2011; Kiers et al., 2011; Weiblen & Treiber, 2015) and the forces that determine levels of specificity or specialization among partners remain poorly understood (Kawakita et al., 2010; Poisot et al., 2011; Wade, 2007). This is especially so for by‐product mutualisms where partners provide benefits that arise as a part of normal life processes and that result in little‐to‐no additional cost to the provider (Connor, 1995).

Currently, many models focus on how mutualisms persist in the face of conflicting interests of the partners. Partners present flexible phenotypes and sanctions or partner choices are exerted upon these phenotypes to weed out cheaters and low return‐on‐investment partners or to enforce reward delivery (Akcay, 2015; Kiers et al., 2011). The mutualisms these models address typically play out against a background of context‐dependency, such that shifts in resource availability or quality either alleviate or enhance conflict among the various phenotypes causing benefits to fluctuate in magnitude and the interaction outcome to slide along a mutualism<>antagonism continuum (Chamberlain et al., 2014; Johnson et al., 1997; Karst et al., 2008). Such models are best applied to mutualisms that involve an exchange of costly rewards and where sufficient variability in partners exists to allow for variability in responses. However, these models provide a poor fit for by‐product mutualisms that, by definition, lack costs associated with the interaction.

**FIGURE 1:**
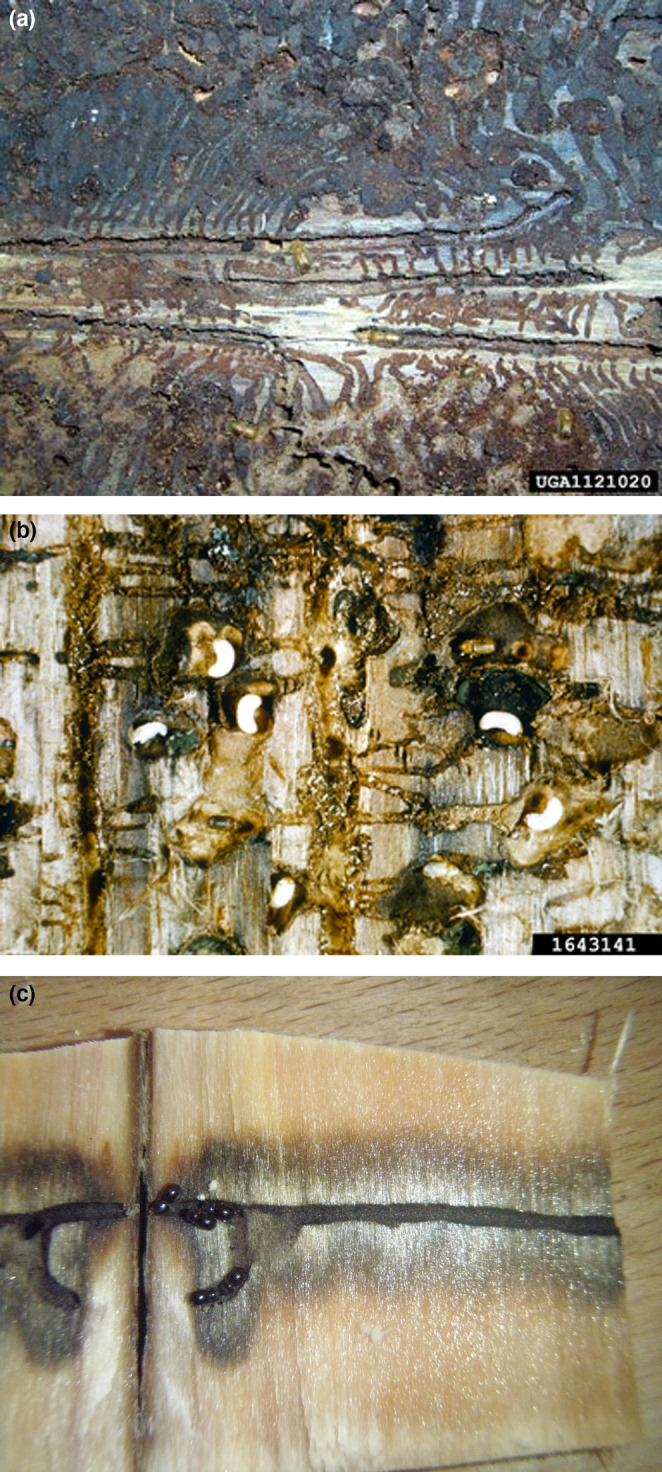
The Scolytinae (bark and ambrosia beetles) (~7500 species) primarily inhabit woody plants and most can be placed into three major ecological types based on their use of plant tissues and fungi. The phloem‐feeding bark beetles (a) do not form close associations with fungi and feed extensively on phloem creating long larval feeding galleries. These beetles lack specialized structures to transport fungi and carry spores incidentally on their integument with high levels of horizontal transmission (Six, 2013). The fungus‐feeding bark beetles (b) are obligately associated with fungi that supplement their phloem diet with nutrients (Adams & Six, 2007; Ayres et al., 2000; Bleiker & Six, 2007; Francke‐Grosmann, 1963; Six & Elser, 2019, 2020; Six & Paine, 1998). These beetles feed and live in phloem but gain a substantial portion of their nutrients from feeding on fungal hyphae in the phloem as larvae and spores in pupal chambers as adults. As a result, larvae consume less of this tree tissue. A very small subset of the fungus‐feeding bark beetles feeds in phloem for only a short period before moving into the even more nutrient‐poor outer bark to complete development feeding only on fungi (Six, 2020b; Six & Elser, 2019). The fungi are transmitted vertically in highly selective fungus‐carrying structures called mycangia (alt. mycetangia; Bleiker et al., 2009; Bracewell & Six, 2015). Ambrosia beetles (c) excavate their tunnels in the sapwood or pith where they feed entirely on fungal layers that form on the tunnel walls, except for a few genera with larvae that feed on fungus‐infested sapwood (Roeper, 1995). Most, but not all, exhibit relatively high specificity with fungi (Batra, 1966; Biedermann & Vega, 2020; Gebhardt et al., 2004; Hulcr & Stelinski, 2017; Kostovcik et al., 2015; Mayers, Harrington, Masuya, et al., 2020). Vertical transmission and specificity are again mediated by mycangia with large complex mycangia being more selective than small less complex ones (Mayers, Harrington, Masuya, et al., 2020; Mayers, Harrington, McNew, et al., 2020). Photo credits: (a) USDA Forest Service, Coeur d'Alene, Bugwood.org, (b) *Daniela Lipastean, University of Suceava*, Bugwood.org *, (*c) Peter Biedermann.

**FIGURE 2:**
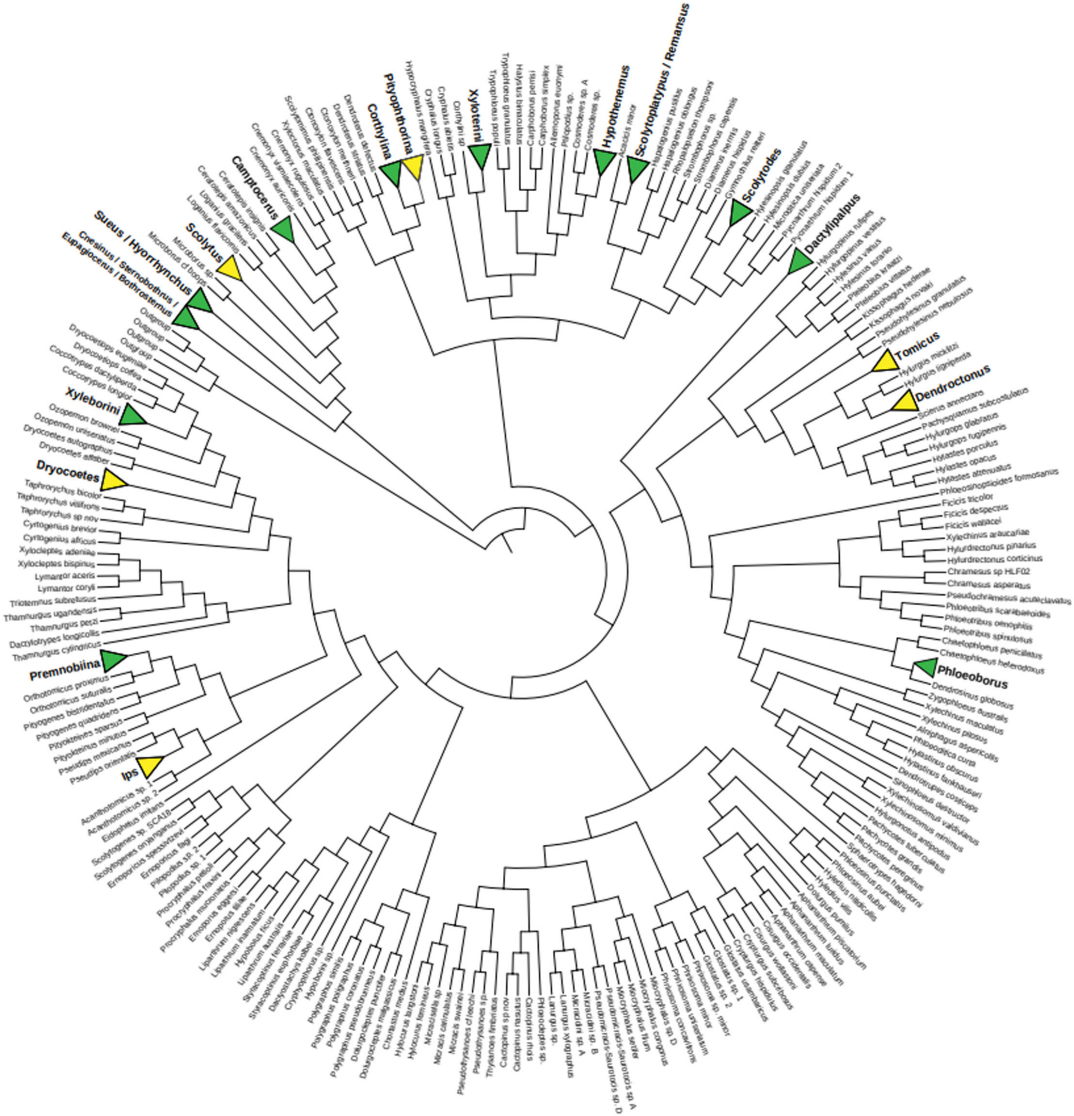
Phylogenetic tree of the Scolytinae showing known independent origins of fungus‐feeding bark beetles (yellow) and ambrosia beetles (green; adapted from Gohli et al., 2017). At least 11 origins of fungus‐feeding are known for ambrosia beetles (Jordal & Cognato, 2012; Kasson et al., 2013) and eight (and potentially 10) for fungus‐feeding bark beetles (Beaver, 1989; Bracewell & Six, 2014; 2015; Bracewell et al., 2018; Farris, 1969; Francke‐Grosmann, 1963; Furniss et al., 1987; Livingston & Berryman, 1972; Mayers et al., 2022; Six & Bentz, 2003; Six & Elser, 2020; Six & Livingston, 2023; Six & Paine, 1996; 1998). Once these mutualisms form, the beetles develop complete dependence on fungi although partner shifts may occur. Reversals from fungus‐feeding to phloem‐feeding are unknown for either type of mutualism. There are no known examples of ambrosia beetles arising from fungus‐feeding bark beetles or vice versa.

Here, we take an approach similar to that of Thrall et al. (2007) who incorporated both resource availability and community complexity to predict the direction, strength, and specificity of symbiotic interactions. In their conceptual model, they developed predictions for the sign (+, 0, −) and specificity of symbioses as a function of environmental quality (productivity) and biotic complexity (diversity and potential for conflict). They predicted that mutualism would be favored when environmental quality is low and antagonism would be favored when environmental quality is high. For mutualisms, they predicted that specificity should be favored when environmental quality is low but biotic complexity is high, and generalism should be favored when both environmental quality and biotic complexity are low. We build on their approach but adjust to a lack of an antagonistic state in by‐product mutualisms. We also allow the use of multiple factors influencing environmental quality, including those exhibiting trade‐offs (defined here as when an increase in one trait is offset by a decrease in another; Garland Jr et al., 2021), rather than presenting environmental quality as a single gradient ranging from high to low. We applied this approach to a large subfamily of weevils (Scolytinae; termed bark and ambrosia beetles) that inhabit woody tissues and that have repeatedly developed two very different evolutionary paths to obligate by‐product mutualisms with fungi (Figure 1 and Figure 2).

Scolytinae‐fungus mutualisms appear to have arisen as a result of the exchange of by‐products (products that occur as part of normal life processes that may or may not be costly to produce but incur little‐to‐no additional cost as the result of the interaction; sensu basal mutualism 1 in Connor, 1995). The fungi gain benefits from reliable transport to an ephemeral resource that occurs as a by‐product of beetle dispersal of spores (Six & Klepzig, 2021), while the beetles, in turn, benefit from nutrient provisioning by the fungi that result from fungal foraging within the tree to support their own reproduction. The fungi are introduced into the tree by the beetles. The fungi then grow out from that entry point and forage for nutrients. The fungi colonize a greater volume of the tree than do the beetles, allowing them to access a larger pool of nutrients. They then translocate nutrients to where larvae are feeding to support their reproduction in the vicinity of their vector (Six & Elser, 2019, 2020). This has the by‐product benefit of providing beetle larvae with sufficient nutrition to complete development (Lehenberger et al., 2021; Six & Elser, 2019; Six & Elser, 2020).

Over time, selection to ensure and enhance benefit delivery can be expected to occur in many by‐product mutualisms (Connor, 1995). In Scolytinae‐fungus mutualisms, simple pits and invaginations of the beetle exoskeleton have evolved into mycangia (specialized structures for fungal transport) that are often highly selective of the fungi they carry. For the fungi, selection over time for greater or more consistent nutrient transport (perhaps through partner choice among low‐ and high‐performing strains) that results in greater host fitness (and in turn, greater fungal fitness) has also likely occurred. Such selection may result in a cost of interaction for one or both partners although the mutualism itself remains based on by‐products (Connor, 1995). For example, the production and maintenance of mycangia ensure contact with a beneficial partner, but evidence suggests they are costly to maintain. In some ambrosia beetles the glands surrounding mycangia degenerate once the fungal garden and brood are established suggesting their maintenance is costly (Schneider & Rudinsky, 1969). However, context dependency in these mutualisms remains extremely low (Six & Klepzig, 2021). While some variation in the magnitude of benefit delivery may occur due to shifts in the environment (e.g., temperature effects on fungal growth, soil nutrients that influence tree quality for fungal foraging) these mutualisms are stable in benefit delivery and never shift from positive to neutral or negative in response to environmental variability (Six & Klepzig, 2021). Low conditionality (small shifts magnitude and no sign change) in these symbioses is not surprising as beetles exploiting marginal substrates can be expected to evolve a high dependence on symbionts required every generation as opposed to symbioses where benefits are needed in only some contexts (e.g., secondary defensive symbionts of insects; Fisher et al., 2017). Indeed, selection is expected to reduce conditionality in obligate mutualisms where symbiont and host fitness interests are strongly aligned (Fisher et al., 2017; Frank, 1997; Sachs et al., 2011).

## 2 THE CONCEPTUAL MODELS

**FIGURE 3:**
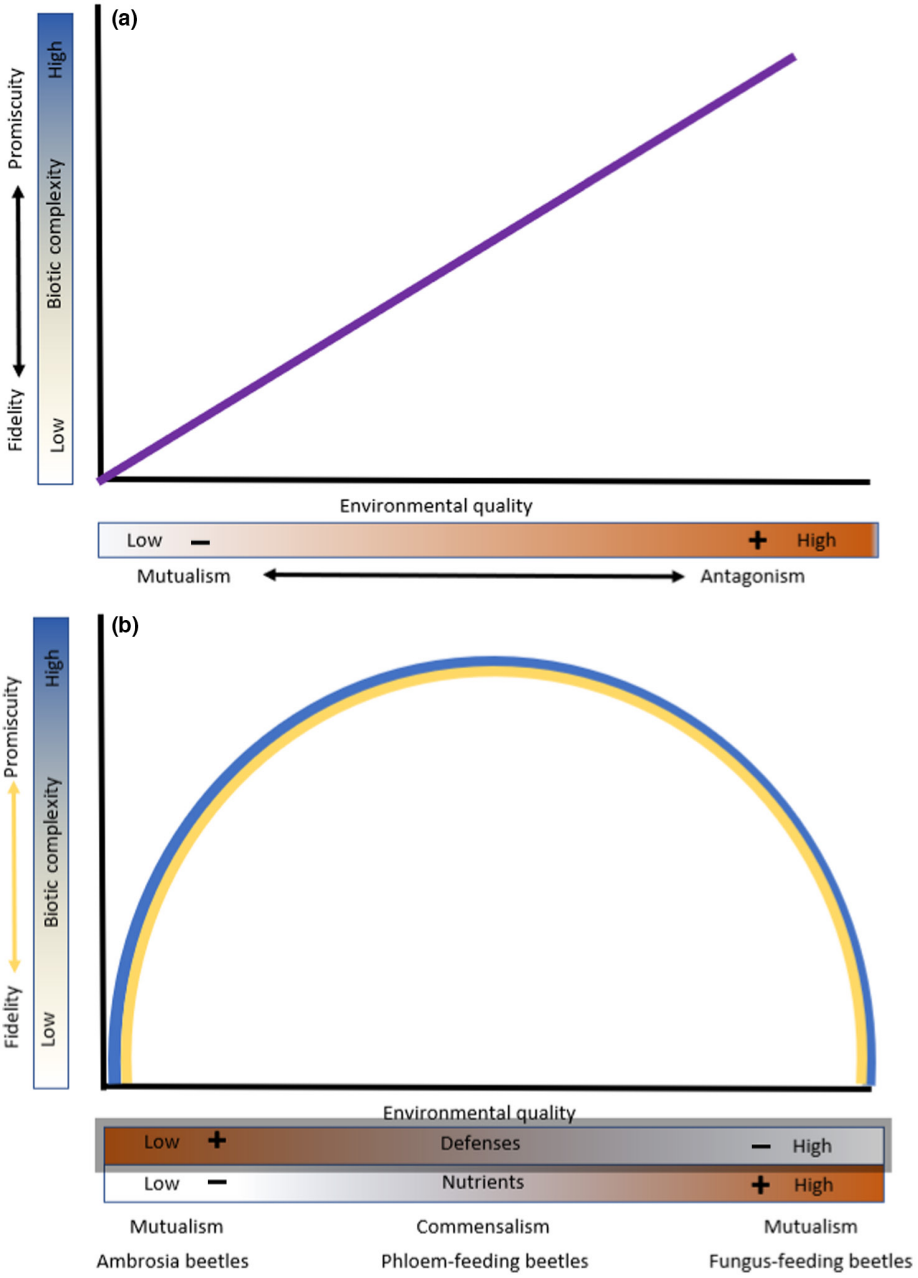
Models of mutualism. (a) In this model (adapted from Thrall et al., 2007), the purple line indicates the predicted outcome of host‐symbiont interactions where, as environmental quality and biotic complexity increase, there is a corresponding increase in generalism, a decrease in fidelity, and an increase in the potential for agonism. (b) In our conceptual model, environmental quality is not presented as a bidirectional force extending from low to high (as in b), but rather as distinct forces that can extend in opposing directions. This allows the inclusion of trade‐offs (increases in the quality of one trait are accompanied by decreases in another) which are common in nature and well‐known to influence interaction outcomes. With the Scolytinae and their fungi, tree nutrient quality, and tree defense produce increasingly strong tradeoffs as one moves away from the middle ground to either the right or left ends of the interaction spectrum. Here, these are shown as gradients from positive to negative (relative to effects on the host beetle). The middle ground (with moderate tree defense and moderate nutrient availability) is occupied by commensalisms (in this case, phloem‐feeding bark beetles). In (b), both the left (ambrosia beetles) and right (fungus‐feeding bark beetles) ends are mutualisms. For beetles to exploit these “ends,” the beetles must partner with high‐functioning fungi. However, because each end presents an opposite set of tradeoffs (either low nutrients (−) and low tree defense (+) or high tree defense (−) and high nutrients (+), the mutualisms that occupy these different ends have developed, and function in fundamentally different ways. Likewise, the mechanisms that lead to, and enforce, increased fidelity and specialization on each end differ. Importantly, Scolytinae‐fungus symbioses cannot be forced to fit the model in (a).

In Figure 3 we contrast the conceptual model developed by Thrall et al. (2007) wherein increases in environmental quality and biotic complexity are expected to lead to increasing promiscuity (generalism) and an enhanced potential for antagonism (Figure 3a) with our conceptual model for by‐product mutualisms using Scolytinae‐fungus symbioses (Figure 3b). We used Thrall et al.'s model as it uses a community approach and provides a clear set of predictions on how biotic complexity and environmental quality interact to affect outcomes that we can compare with the results of our alternate model.

For background, the symbiosis types found in Scolytinae are described in Figure 1. In Figure 2, we present a phylogeny indicating the positions of the known independent origins of the two mutualism types out of non‐fungus‐dependent phloem‐feeding beetles. Table 1 lists the known obligate Scolytinae‐fungus mutualisms.

**TABLE 1:** Independent origins of obligate mutualisms with fungi in Scolytinae.

	Clade	Number of known species
Obligate mutualism in bark beetles	*Dendroctonus*	Many
	*Dryocoetes*	1; *Dryocoetes confusus*
	*Tomicus*	1; *Tomicus minor*
	*Scolytus*	1; *Scolytus ventralis*
	*Ips*	At least two
	*Pityoborus*	Many
Obligate mutualism in ambrosia beetles	Xyleborini (tribe)	All species
	Premnobiina (subtribe)	All species
	*Hypothenemus*	1; *Hypothenemus curtipennis*
	Xyloterini (tribe)	All species
	Corthylini (tribe)	All species
	*Sueus/Hyorrhynchus*	All species
	*Cnesinus/Bothrosternus/Eupagiocerus/Sternobothrus*	All species
	*Scolytoplatypus/Remansus*	All species
	*Scolytodes*	1; *Scolytodes unipunctatus*
	*Camptocerus*	All species
	*Phloeoborus*	All species
	*Dactylipalpus*	All species

*Note*: The number of known species per clade with obligate mutualisms is given. Clades are either represented by single species, genera, subtribes, or tribes. The Scolytinae are greatly under‐sampled for mutualism and likely many more exist.

## 3 INTERPRETING OUR CONCEPTUAL MODEL

The interactions between Scolytinae beetles and fungi exist on a mutualism<>commensalism<>mutualism spectrum (Figure 3), rather than on a mutualism<> commensalism<>antagonism spectrum. *The middle* of the spectrum is the domain of phloem‐feeding bark beetles (non‐fungus dependent), the basal feeding strategy from which all fungus‐feeding lineages have arisen (Kirkendall et al., 2015; Figure 2). Phloem‐feeding bark beetles use weak, dying, or recently killed hosts. Here, reduced plant/tree defenses coupled with moderate nutrient quality create an environment of “easy pickings” resulting in high biotic diversity of beetles and fungi, and high promiscuity. This leads to competition which we suggest is the ultimate driving force for the development of the two different pathways to mutualism that allow exploitation of the more “challenging” right and left ends of the spectrum.

Differences in tradeoffs in environmental quality (tree defenses and nutritional quality) associated with the two ends of the spectrum determine the mutualism outcome (Figure 3). *On the right end*, we find the tree‐killing fungus‐feeding bark beetles. Here, strong defenses in tree hosts trump the benefit of the higher levels of nutrients found in healthy fresh phloem and act to severely restrict biotic complexity as both beetles and fungi must be adapted to tree defenses and to survive in an initially living host. Because these beetles must mass attack to overcome tree defenses (Lindgren & Raffa, 2013), intra‐specific competition for phloem is high and nutrient provisioning by fungi is crucial to support developing broods in a densely populated resource. Feeding galleries of the larvae of these beetles are short indicating a high dependence on fungi for nutrients (Figure 1). Here, only fungi that provide adequate nutrient provisioning can act as partners, further reducing biotic complexity and supporting the formation of highly specific partnerships with high‐functioning fungal partners. Conversely, *at the left end*, the domain of ambrosia beetles, the low nutrient quality of pith or sapwood of dead/dying trees trumps the benefit of low‐to‐no defenses, again restricting biotic complexity. In such trees, low nutrient availability in sapwood constrains beetle and fungal communities and requires beetles to partner only with fungi capable of providing a complete diet further acting to decrease biotic complexity and enforcing greater specificity and fidelity.

In both the right and left ends of the spectrum mutualisms with fungi allow Scolytinae to exploit otherwise intractable niches. *However, because each niche imposes an opposite set of tradeoffs, the partnerships function in fundamentally different ways. Likewise, the mechanisms that lead to and enforce increased fidelity in each case differ*.

## 4 LIFE ON THE SPECTRUM: PROMISCUITY OR FIDELITY?

And if you can't be with the one you love honey, love the one you're with—Stephen Stills

You're my dream come true, my one and only you—Buck Ram

Here, we describe in greater detail how differences in biotic complexity (symbiont pools, other bark beetles, numbers of tree hosts) and tradeoffs in the major factors influencing environmental quality (tree defenses and nutritional quality) result in different mutualism pathways in our model and a corresponding gradient of partner specialization extending from highly promiscuous to commensal or facultative “friends‐with‐benefits” to fidelity.

### 4.1 Life in the middle

The phloem‐feeding strategy is found in the oldest lineages within the Scolytinae and is basal to all known origins of fungus‐feeding bark and ambrosia beetles (Kirkendall et al., 2015). Phloem‐feeding beetles appear to compensate for a lack of nutrient provisioning by fungi by consuming large amounts of phloem as larvae (Figure 1) which can lead to high levels of intra‐ and inter‐specific competition. The beetles and fungi are relatively generalized which acts to increase biotic complexity. Several species often co‐colonize trees and compete for resources in weak, dying, or recently dead trees, twigs, or branches. Because these substrates have lower defenses than healthy trees, few of these beetles employ pheromone‐mediated mass attacks. Also, beetles do not need specialized detoxification systems and there is less specificity to particular host tree species increasing the host tree range. For these beetles, intra‐ and inter‐specific competition depends mainly on host tree condition, availability, and background beetle population levels (Anderbrandt et al., 1985; Pineau et al., 2017). For those that do employ aggregation pheromones, attack densities can be very high (e.g., *Ips typographus*) leading to high levels of intra‐specific competition with strong negative effects on fitness (Schlyter & Birgersson, 1985).

While all phloem‐feeding bark beetles vector and develop with fungi, their fungal communities are variable and horizontally transmitted. Many of the fungi are found with several beetle species, especially those that co‐colonize the same trees (e.g., Kirisits, 2007; Kirschner, 2001; Kolařík et al., 2017; Kolarik & Jankowiak, 2013). The prevalence of these fungi in and among beetle populations is largely driven by abiotic conditions, geography, and host tree phylogeny (Kirisits, 2007, Kirschner, 2001; Kolařík et al., 2017). Many are likely commensals, exploiting the beetle for transport to new substrates, but with little effect on the host beetle (Birkemoe et al., 2018; Seibold et al., 2019). Some may be antagonists that compete for nutrients or facultative mutualists that provide some degree of nutritional benefit or detoxification of tree‐defensive chemistry *when they are present*. However, because these “friends with benefits” *are often absent*, they cannot reliably provide benefits and host beetles must be able to survive independent of their presence.

### 4.2 Why leave the middle ground?

Phloem‐feeding beetles and fungi experience greater biotic complexity as they use a broader range of tree species and are often exposed to other beetles and their symbiont pools. In such a constantly shifting milieu, horizontal transmission of environmentally acquired fungi disrupts the potential for a shared fate while increasing the potential for competition among symbionts and exploitation of the host (Fisher et al., 2017). Beetles in the middle ground are essentially “on their own” to gain the nutrients they require, and the fungi are open to access any number of beetle vectors for dispersal.

The strategy to go it alone or to “love the one you are with” in the case of facultative benefits, has clearly been successful for many Scolytinae and fungi. However, the multiple independent origins of fungus‐feeding (Figure 2) suggest there are also substantial advantages to specialize in high‐functioning partners. Competition in the middle ground appears to have been the driving force for some beetles and fungi to move from promiscuity to “tying the knot” so they could exploit the more challenging but relatively empty niche spaces of the right and left ends of the spectrum. The mutualisms that formed have persisted for long periods of time (Bracewell et al., 2018; Six, 2020a; Vanderpool et al., 2017) and are also resistant, although not impervious, to partner‐swapping and divorce (Mayers et al., 2022; Six, 2020b). Additionally, reversals to promiscuity and strict phloem‐feeding have not been observed indicating that once dependence and specialization develop, partner and strategy shifts are highly constrained.

### 4.3 Life on the right

All known fungus‐feeding bark beetles are tree‐killing conifer‐colonizing species. These beetles live in the best nutritional resources (fresh phloem, at least initially, of relatively healthy trees), but with the tradeoff that they are also those most hostile because of strong tree defenses. To overcome defenses and kill the tree, these beetles must attack *en masse*. However, mass attack results in a tradeoff between the number of beetles needed to kill the tree and the amount of phloem resources available for brood. The fungi alleviate this problem by foraging throughout the sapwood via the ray parenchyma where they acquire nitrogen and phosphorus that they in turn translocate to the phloem to support their own reproduction in a place where they are assured their vectors will acquire their spores for dispersal (pupal cradles). A by‐product of nutrient provisioning is that the increase in concentrations of limiting nutrients in the phloem reduces the amount of phloem required by each larva and the high densities of brood resulting from large numbers of mass‐attacking adults can be accommodated. Early entry into the tree reduces exposure to other bark beetles and fungi by allowing them to colonize and capture space in a relatively “empty” tree and to use high‐nutrient quality phloem.

Fungus‐feeding beetles and their fungi are specialized to use particular tree species due to adaptation to secondary chemistry. This narrows the potential for inter‐specific competition with other tree‐killing bark beetles and fungi. This also restricts the size of the symbiont pools to which they are exposed, facilitating their ability to develop and maintain stable associations with efficient partners. The evolution of selective mycangia (complex transport structures) by these beetles to facilitate vertical transmission and the reduction of sexual reproduction by the mutualist fungi, most of which are predominantly asexual, has further enforced fidelity among these beetles and their fungal partners.

### 4.4 Life on the left

Ambrosia beetles live in sapwood, a substrate notoriously low in nutrients (Filipiak & Weiner, 2014; Lehenberger et al., 2021). This requires beetles to consort with fungi capable of meeting all their nutritional needs and adapted to living and foraging in this difficult substrate. The majority of these fungi are primary foragers like those associated with bark beetles (Lehenberger et al., 2019). Like fungus‐feeding bark beetles, ambrosia beetles also possess selective mycangia that support the vertical transmission of their fungi (Francke‐Grosmann, 1963; Mayers et al., 2022). Entry by beetles and their fungi into sapwood typically occurs early in the decline or death of a tree, and in most cases, only into wood containing ethanol (produced in response to stressors) and when few other fungi exist as potential competitors (Ranger et al., 2018). Moreover, ethanol acts as a biological filter to select specific fungal mutualists because of the competitive advantages of the ethanol‐resistant and ethanol‐consuming cultivars (Ranger et al., 2018). This combination of early colonization ahead of wood‐rotting saprophytes, biological filtering by ethanol, and the difficulty of making a living in this substrate by both beetles and fungi acts to substantively reduce the host and symbiont pools to which they are exposed enhancing their ability to form stable associations.

Unlike bark beetles whose larvae feed independently of one another, ambrosia beetles feed communally, feeding on fungi growing on gallery walls and displaying grooming behaviors that reduce loads of fungal antagonists (Diehl et al., 2022). Furthermore, some species occupy communal nests for long periods and produce multiple generations allowing the use of established cultivars over time. This continuous contact is further enhanced by inbreeding and, in some, sociality and fungus‐farming (Diehl et al., 2022; Nuotclà et al., 2019, 2021). The most advanced social farmers are found in the most durable and early colonized wood substrates, a situation that greatly supports the establishment and maintenance of the mutualist fungi (Biedermann & Rohlfs, 2017; Diehl et al., 2022; Nuotclà et al., 2019). Community filtering via ethanol, fungus farming, and asexuality in the symbionts further supports the most stable and specific beetle‐fungus associations in the most social species.

### 4.5 Lessons learned

Removing the potential for a shift to an alternate neutral or antagonistic state and allowing the use of multiple factors influencing environmental quality rather than combining them into a single gradient of quality ranging from high to low allowed us to modify the Thrall et al. (2007) model in a manner that allowed a fit with Scolytinae‐fungus mutualisms and that can describe the two pathways to mutualisms that have repeatedly evolved in this group of beetles. With these adjustments, we asked how well our model agreed with the predictions of the original model. Thrall et al. (2007) defined environmental quality as productivity, and we assumed this to include factors that affect access to productivity such as plant defenses. Our incorporation of trade‐offs in environmental quality posed some challenges in assessing their prediction that mutualism should be favored when environmental quality is low and antagonism favored when environmental quality is high. For fungus‐feeding bark beetles, nutrient quality is high, but high defenses make them difficult to access. For ambrosia beetles, low defenses make for a more benign environment, but nutrients are limiting. However, stepping back, the tradeoffs themselves may be seen as lowering environmental quality because they create a more challenging situation in comparison to the middle ground where nutrient and tree defense levels are both moderate. From this view, the prediction of mutualism being favored when environmental quality is low is met. The incorporation of the trade‐offs in environmental quality helped define overall environmental quality in this system as well as providing explanatory power on why the two mutualisms are fundamentally different.

Our model did not agree with Thrall et al.'s prediction that mutualism specificity should be favored when environmental quality is low and biotic complexity is high and generalism should be favored when both environmental quality and biotic complexity are low. They argue that hosts should evolve greater specificity in response to increasing diversity in mutualists; however, it can also be posited that greater symbiont diversity reduces the ability of a host and symbiont to reliably interact and form a stable association. In Scolytinae‐fungus symbioses, generalism occurs in the middle ground which is exemplified by the highest environmental quality (i.e., best on the spectrum relative to the effect of tradeoffs on the ends) and the highest biotic complexity, while the greatest specificity occurred on the right and left ends of the spectrum where both environmental quality and biotic complexity were lowest. This makes sense in that mutualists are often required to exploit difficult environments and often require specific mutualists possessing particular traits. Thrall et al. (2007), however, did recognize that more diverse communities may present more potential for conflict and that the effectiveness of particular mutualisms may decline as diversity increases.

In the case of our system, the characteristics of the left and right ends of the spectrum apply several filters (host tree specialization and tree defenses in the case of fungus‐feeding bark beetles, and ethanol and low nutrient quality in the case of ambrosia beetles) that lower biotic diversity making the formation of consistent associations among high‐functioning partners more likely. Further, vertical transmission of the fungi by the beetles also supports greater consistency of partners for reliable benefit delivery. The environments these mutualisms inhabit are also more predictable and stable than those found in the middle ground. High symbiont reliability, low symbiont richness, and high environmental predictability are expected to reduce context dependency (Thrall et al., 2007) as we have found in the fungus‐feeding bark beetles and ambrosia beetles (Six & Klepzig, 2021).

## 5 THE VALUE OF “FLEXIBLE” MODELS OF MUTUALISM

Trying to apply most existing models to by‐product mutualisms can be difficult. These mutualisms do not fit traditional models based on partner conflict. They do not have agonistic alternate states and do not lend themselves to models invoking the tragedy of the commons or sanctions. To be inclusive of the range of diversity in mutualisms, including by‐products, will require some flexibility to account for differences in interaction drivers and outcomes.

When looking at specific mutualisms, we argue that there is value in presenting environmental quality, not as a bidirectional axis extending from low to high, but rather as separate axes for each main factor that influences quality to allow the incorporation of tradeoffs when they exist. These tradeoffs may be crucial to understanding interaction outcomes but are obscured when environmental factors are combined. Incorporating such tradeoffs into our model was crucial in explaining the development of the two fundamentally different mutualisms.

## AUTHOR CONTRIBUTIONS

**Peter H. W. Biedermann:** Project administration (equal); writing – review and editing (equal). **Diana L. Six:** Conceptualization (lead); project administration (equal); writing – original draft (lead); writing – review and editing (equal).

## FUNDING INFORMATION

A personal meeting of the authors was enabled through a Mercator fellowship to DS as part of the research unit FOR 5375 funded by the Deutsche Forschungsgemeinschaft (DFG, German Research Foundation)—459717468 (sub‐project 2: 500516919). PB acknowledges the support of the DFG in the framework of his Emmy Noether project BI 1956/1–1.

## Data Availability

No data were generated for this paper.

## References

[ece310345-bib-0001] Adams, A. S. , & Six, D. L. (2007). Temporal variation in mycophagy and prevalence of fungi associated with developmental stages of *Dendroctonus ponderosae* (Coleoptera: Curculionidae). Environmental Entomology, 36, 64–72. 10.1093/ee/36.1.64 17349118

[ece310345-bib-0002] Akcay, E. (2015). Evolutionary models of mutualism. In J. L. Bronstein (Ed.), Mutualism (pp. 57–76). Oxford University Press.

[ece310345-bib-0003] Anderbrandt, O. , Schlyter, F. , & Birgersson, G. (1985). Intraspecific competition affecting parents and offspring in the bark beetle *Ips typographus* . Oikos, 53, 89–98. 10.2307/3565226

[ece310345-bib-0005] Ayres, M. P. , Wilkens, R. T. , Ruel, J. J. , Lombardero, M. J. , & Vallery, E. (2000). Nitrogen budgets of phloem‐feeding bark beetles with and without symbiotic fungi. Ecology, 81, 2198–2210. 10.1890/0012-9658

[ece310345-bib-0006] Batra, L. R. (1966). Ambrosia fungi: Extent of specificity to ambrosia beetles. Science, 153, 193–195. 10.1126/science.153.3732.193 17831508

[ece310345-bib-0007] Beaver, R. A. (1989). Insect–fungus relationships in the bark and ambrosia beetles. In N. Wilding , N. M. Collins , P. M. Hammond , & J. F. Webber (Eds.), Insect–fungus interactions (pp. 121–143). Academic Press.

[ece310345-bib-0008] Biedermann, P. H. , & Rohlfs, M. (2017). Evolutionary feedbacks between insect sociality and microbial management. Current Opinion in Insect Science, 22, 92–100. 10.1016/j.cois.2017.06.003 28805645

[ece310345-bib-0009] Biedermann, P. H. , & Vega, F. E. (2020). Ecology and evolution of insect–fungus mutualisms. Annual Review of Entomology, 65, 431–455. 10.1146/annurev-ento-011019-024910 31610133

[ece310345-bib-0010] Birkemoe, T. , Jacobsen, R. M. , Sverdrup‐Thygeson, A. , & Biedermann, P. H. (2018). Insect‐fungus interactions in dead wood systems. In M. Ulyshen (Ed.), Saproxylic insects (pp. 377–427). Springer.

[ece310345-bib-0011] Bleiker, K. , & Six, D. L. (2007). Dietary benefits of fungal associates to an eruptive herbivore: Potential implications of multiple associates on host population dynamics. Environmental Entomology, 36, 1384–1396. 10.1093/ee/36.6.1384 18284766

[ece310345-bib-0012] Bleiker, K. P. , Potter, S. E. , Lauzon, C. R. , & Six, D. L. (2009). Transport of fungal symbionts by mountain pine beetle. Canadian Entomologist, 141, 505–514. 10.4039/n09-034

[ece310345-bib-0013] Borges, R. M. (2017). Co‐niche construction between hosts and symbionts: Ideas and evidence. Journal of Genetics, 96, 483–489. 10.1007/s12041-017-0792-9 28761011

[ece310345-bib-0014] Bourke, A. F. G. (2011). Principles of Social Evolution (pp. 280). Oxford University Press. doi:10.1016/j.baae.2012.01.003

[ece310345-bib-0015] Bracewell, R. R. , & Six, D. L. (2014). Broadscale specificity in a bark beetle‐fungal symbiosis: A spatio‐temporal analysis of the mycangial fungi of the western pine beetle. Microbial Ecology, 68, 859–870. 10.1007/s00248-014-0449-7 25004995

[ece310345-bib-0016] Bracewell, R. R. , & Six, D. L. (2015). Experimental evidence of bark beetle adaptation to a fungal symbiont. Ecology and Evolution, 5, 5109–5119. 10.1002/ece3.1772 26640686 PMC4662301

[ece310345-bib-0017] Bracewell, R. R. , Vanderpool, D. , Good, J. , & Six, D. L. (2018). Cascading speciation among mutualists and antagonists in a tree‐beetle‐fungal interaction. Proceedings of the Royal Society. B, 285, 20180694. 10.1098/rspb.2018.0694 30051849 PMC6030525

[ece310345-bib-0018] Chamberlain, S. A. , Bronstein, J. L. , & Rudgers, J. A. (2014). How context dependent are species interactions? Ecology Letters, 17, 881–890. 10.1111/ele.12279 24735225

[ece310345-bib-0019] Connor, R. C. (1995). The benefits of mutualism: A conceptual framework. Biological Reviews, 70, 427–457. 10.1111/j.1469-185X.1995.tb01196.x

[ece310345-bib-0020] Diehl, J. M. C. , Kowallik, V. , Keller, A. , & Biedermann, P. H. W. (2022). First experimental evidence for active fungus farming in ambrosia beetles and strong heredity of garden microbes. Proceedings of the Royal Society. B, 289, 20221458. 10.1098/rspb.2022.1458 36321493 PMC9627711

[ece310345-bib-0021] Farris, S. H. (1969). Occurrence of mycangium in the bark beetle *Dryocoetes confusus* (Coleoptera: Scolytidae). Canadian Entomologist, 101, 527–532. 10.4039/Ent101527-5

[ece310345-bib-0022] Filipiak, M. , & Weiner, J. (2014). How to build a beetle out of wood: Multielemental stoichiometry of wood decay, xylophagy, and fungivory. PLoS One, 9, 1151104. 10.1371/journal.pone.0115104 PMC4275229 25536334

[ece310345-bib-0023] Fisher, R. M. , Henry, L. M. , Cornwallis, C. K. , Kiers, E. T. , & West, S. A. (2017). The evolution of host‐symbiont dependence. Nature Communications, 8, 15973. 10.1038/ncomms15973 PMC5500886 28675159

[ece310345-bib-0024] Francke‐Grosmann, H. (1963). Some new aspects in forest entomology. Annual Review of Entomology, 8, 415–438. 10.1146/annurev.en.08.010163.002215

[ece310345-bib-0025] Frank, S. A. (1997). Models of symbiosis. The American Naturalist, 150, S80–S99. 10.1086/286051 18811314

[ece310345-bib-0026] Furniss, M. M. , Woo, J. Y. , Deyrup, M. A. , & Atkinson, T. H. (1987). Prothoracic Mycangium on pine‐infesting *Pityoborus* spp. (Coleoptera: Scolytidae). Annals of the Entomological Society of America, 80, 692–696. 10.1093/aesa/80.5.692

[ece310345-bib-0027] Garland, T., Jr. , Downs, C. L. , & Ives, A. R. (2021). Trade‐offs (and constraints) in organismal biology. Physiological and Biochemical Zoology, 95, 82–112. 10.1086/717897 34905443

[ece310345-bib-0028] Gebhardt, H. , Begerow, D. , & Oberwinkler, F. (2004). Identification of the ambrosia fungus of *Xyleborus monographus* and *X. dryographus* (Coleoptera: Curculionidae, Scolytinae). Mycological Progress, 3, 95–102. 10.1007/s11557-006-0080-1

[ece310345-bib-0029] Gohli, J. , Kirkendall, L. R. , Smith, S. M. , Cognato, A. I. , Hulcr, J. , & Jordahl, B. H. (2017). Biological factors contributing to bark and ambrosia beetle species diversification. Evolution, 71, 1258–1272. 10.1111/evo.13219 28257556

[ece310345-bib-0032] Hulcr, J. , & Stelinski, L. L. (2017). The ambrosia symbiosis: From evolutionary ecology to practical management. Annual Review of Entomology, 6, 285–303. 10.1146/annurev-ento-031616-035105 27860522

[ece310345-bib-0034] Johnson, N. C. , Graham, J. H. , & Smith, F. A. (1997). Functioning of mycorrhizal associations along the mutualism‐parasitism continuum. New Phytologist, 135, 575–586.

[ece310345-bib-0035] Jordal, B. H. , & Cognato, A. I. (2012). Molecular phylogeny of bark and ambrosia beetles reveals multiple origins of fungus farming during periods of global warming. Evolutionary Biology, 12, 133. 10.1186/1471-2148-12-133 22852794 PMC3514184

[ece310345-bib-0037] Karst, J. , Marczak, L. , Jones, M. D. , & Turkington, R. (2008). The mutualism‐parasitism continuum in ectomycorrhizas: A quantitative assessment using meta‐analysis. Ecology, 89, 1032–1042. 18481528 10.1890/07-0823.1

[ece310345-bib-0038] Kasson, M. T. , O'Donnell, K. , Rooney, A. P. , Sink, S. , Ploetz, J. N. , Konkol, J. L. , Konkol, J. L. , Carrillo, D. , Freeman, S. , Mendel, Z. , Smith, J. A. , Black, A. W. , Hulcr, J. , Bateman, C. , Stefkova, K. , Campbell, P. R. , Geering, A. D. , Dann, E. K. , Eskalen, A. , … Geiser, D. M. (2013). An inordinate fondness for *Fusarium*: Phylogenetic diversity of fusaria cultivated by ambrosia beetles in the genus *Euwallacea* on avocado and other host plants. Fungal Genetics and Biology, 56, 147–157. 10.1016/j.fgb.2013.04.004 23608321

[ece310345-bib-0040] Kawakita, A. , Okamoto, T. , Goto, R. , & Kato, M. (2010). Mutualism favors higher host specificity than does antagonism in plant‐herbivore interaction. Proceedings of the Royal Society. B, 277, 2765–2774. 10.1098/rspb.2010.0355 20427340 PMC2981980

[ece310345-bib-0041] Kiers, E. T. , Duhamel, M. , Beesetty, Y. , Mensah, J. A. , Franken, O. , Verbruggen, E. , Fellbaum, C. R. , Kowalchuk, G. A. , Hart, M. M. , Bago, A. , Palmer, T. M. , West, S. A. , Vandenkoornhuyse, P. , Jansa, J. , & Bücking, H. (2011). Reciprocal rewards stabilize cooperation in the mycorrhizal symbiosis. Science, 333(6044), 880–882. 21836016 10.1126/science.1208473

[ece310345-bib-0042] Kirisits, T. (2007). Fungal associates of European bark beetles with special emphasis on the ohiostomatoid fungi. In F. Lieutier , F. Day , K. R. Batisti , J. C. Greigoire , & H. F. Evans (Eds.), Bark and wood‐boring insects in living trees in Europe, a synthesis (pp. 181–236). Kluwer Academic Publishers.

[ece310345-bib-0043] Kirkendall, L. R. , Biederman, P. H. W. , & Jordal, B. H. (2015). Evolution and diversity of bark and ambrosia beetles. In F. E. Vega & R. W. Hofstetter (Eds.), Bark beetles: Biology and ecology of native and invasive beetles (pp. 85–156). Academic Press. 10.1016/B978-0-12-417156-5.00003-4

[ece310345-bib-0044] Kirschner, R. (2001). Diversity of filamentous fungi in bark beetle galleries in Central Europe. In J. K. Misra (Ed.), Trichomycetes and other fungal groups (pp. 175–196). CRC Press.

[ece310345-bib-0045] Kolařík, M. , Hulcr, J. , Tisserat, N. , DeBeer, W. , Stovcik, M. , & Kolarikova, Z. (2017). *Geosmithia* associated with bark beetles and woodborers in the western USA: Taxonomic diversity and vector specificity. Mycologia, 109, 185–199. 10.1080/00275514.2017.1303861 28448771

[ece310345-bib-0046] Kolarik, M. , & Jankowiak, R. (2013). Vector affinity and diversity of *Geosmithia* fungi living on subcortical insects inhabiting Pinaceae species in central and northeastern Europe. Microbial Ecology, 66, 682–700. 10.1007/s00248-013-0228-x 23624540

[ece310345-bib-0049] Kostovcik, M. , Bateman, C. C. , Kolarik, M. , Stelinski, L. L. , Jordal, B. H. , & Hulcr, J. (2015). The ambrosia symbiosis is specific in some species and promiscuous in others: Evidence from community pyrosequencing. The ISME Journal, 9, 126–138. 10.1038/ismej.2014.115 25083930 PMC4274425

[ece310345-bib-0050] Lehenberger, M. , Biedermann, P. H. , & Benz, J. P. (2019). Molecular identification and enzymatic profiling of *Trypodendron* (Curculionidae: Xyloterini) ambrosia beetle‐associated fungi of the genus *Phialophoropsis* (Microascales: Ceratocystidaceae). Fungal Ecology, 38, 89–97. 10.1016/j.funeco.2018.07.010

[ece310345-bib-0051] Lehenberger, M. , Foh, N. , Gottlein, A. , Six, D. L. , & Biedermann, P. H. W. (2021). Nutrient‐poor breeding substrates of ambrosia beetles are enriched with biologically important elements. Frontiers in Microbiology, 12, 664542. 10.3389/fmicb.2021.664542 33981292 PMC8107399

[ece310345-bib-0052] Lindgren, B. S. , & Raffa, K. F. (2013). Evolution of tree killing in bark beetles (Coleoptera: Curculionidae): Trade‐offs between the maddening crowds and a sticky situation. The Canadian Entomologist, 145, 471–495. 10.4039/tce.2013.27

[ece310345-bib-0053] Livingston, R. L. , & Berryman, A. A. (1972). Fungus transport structures in the fir engraver, *Scolytus vertralis* (Coleoptera: Scolytidae). The Canadian Entomologist, 104, 1793–1800.

[ece310345-bib-0055] Mayers, C. G. , Harrington, T. C. , & Biedermann, P. H. (2022). Mycangia define the diverse ambrosia beetle–fungus symbioses. In T. R. Schultz , R. Gawne , & P. N. Peregrine (Eds.), The convergent evolution of agriculture in humans and insects (pp. 105–142). The MIT Press.

[ece310345-bib-0056] Mayers, C. G. , Harrington, T. C. , Masuya, H. , Jordal, B. H. , McNew, D. L. , Shih, H. H. , Roets, F. , & Kietzka, G. J. (2020). Patterns of coevolution between ambrosia beetle mycangia and the Ceratocystidaceae, with five new fungal genera and seven new species. Persoonia, 44, 41–66. 10.3767/persoonia.2020.44.02 33116335 PMC7567963

[ece310345-bib-0057] Mayers, C. G. , Harrington, T. C. , McNew, D. L. , Roeper, R. R. , Biedermann, P. H. W. , Masuya, H. , & Bateman, C. C. (2020). Four mycangial types and four genera of ambrosia fungi suggest a complex history of fungus‐farming in the ambrosia beetle tribe Xyloterini. Mycologia, 112, 1104–1137. 10.1080/00275514.2020.1755209 32552515

[ece310345-bib-0058] Nuotclà, J. A. , Biedermann, P. H. , & Taborsky, M. (2019). Pathogen defence is a potential driver of social evolution in ambrosia beetles. Proceedings of the Royal Society B, 286, 20192332. 10.1098/rspb.2019.2332 31847779 PMC6939916

[ece310345-bib-0059] Nuotclà, J. A. , Diehl, J. M. C. , & Taborsky, M. (2021). Habitat quality determines dispersal decisions and fitness in a beetle–fungus mutualism. Frontiers in Ecology and Evolution, 9, 602672. 10.3389/fevo.2021.602672

[ece310345-bib-0061] Pineau, X. , Bourguignon, M. , Jactel, H. , Lieutier, F. , & Salle, A. (2017). Pyrrhic victory for bark beetles: Successful standing trees colonization triggers strong intra‐specific competition for offspring of *Ips sexdentatus* . Forest Ecology and Management, 99, 188–196. 10.1016/j.foreco.2017.05.044

[ece310345-bib-0062] Poisot, T. , Bever, J. D. , Nemri, A. , Thrall, P. , & Hochberg, M. E. (2011). A conceptual framework for the evolution of specialization. Ecology Letters, 14, 841–851. 10.1111/j.1461-0248.2011.01645.x 21699641 PMC3152695

[ece310345-bib-0063] Ranger, C. , Biedermann, P. H. W. , Phuntumart, V. , Beligala, G. U. , Ghosh, S. , Palmquist, D. E. , Mueller, R. , Barnett, J. , Schultz, P. B. , Reding, M. E. , & Benz, J. P. (2018). Symbiont selection via alcohol benefits fungus farming by ambrosia beetles. Proceedings of the National Academies of Science of the United States of America, 115, 4447–4452. 10.1073/pnas.171685211 PMC5924889 29632193

[ece310345-bib-0064] Roeper, R. A. (1995). Patterns of mycetophagy in Michigan ambrosia beetles. Michigan Academian, 27, 153–161.

[ece310345-bib-0065] Sachs, J. L. , Essenberg, C. J. , & Turcotte, M. M. (2011). New paradigms for the evolution of beneficial infections. Trends in Ecology and Evolution, 26, 202–209. 10.1016/j.tree.2011.01.010 21371775

[ece310345-bib-0066] Schneider, I. A. , & Rudinsky, J. A. (1969). Mycetangial glands and their seasonal changes in *Gnathotrichus retusus* and *G. sulcatus* . Annals of the Entomological Society of America, 62, 39–43.

[ece310345-bib-0067] Seibold, S. , Müller, J. , Baldrian, P. , Cadotte, M. W. , Štursová, M. , Biedermann, P. H. , Krah, F. S. , & Bässler, C. (2019). Fungi associated with beetles dispersing from dead wood–Let's take the beetle bus! Fungal Ecology, 39, 100–108. 10.1016/j.funeco.2018.11.016

[ece310345-bib-0068] Six, D. L. (2013). The bark beetle holobiont: Why microbes matter. Journal of Chemical Ecology, 39, 989–1002. 10.1007/s10886-013-0318-8 23846183

[ece310345-bib-0069] Six, D. L. (2020a). Niche construction theory can link bark beetle‐fungus symbiosis type and colonization behavior to large‐scale causal chain effects. Current Opinion in Insect Science, 39, 27–34. 10.1016/j.cois.2019.12.005 32114295

[ece310345-bib-0070] Six, D. L. (2020b). A major symbiont shift supports a major niche shift in a clade of tree‐killing bark beetles. Ecological Entomology, 45, 190–201. 10.1111/een.12786

[ece310345-bib-0071] Six, D. L. , & Bentz, B. J. (2003). The fungi associated with the north American spruce beetle, *Dendroctonus rufipennis* . Canadian Journal of Forest Research, 33, 1815–1820. 10.1139/X03-107

[ece310345-bib-0072] Six, D. L. , & Elser, J. J. (2019). Extreme ecological stoichiometry of a bark beetle‐fungus mutualism. Ecological Entomology, 44, 543–551. 10.1111/een.12731

[ece310345-bib-0073] Six, D. L. , & Elser, J. J. (2020). Mutualism is not restricted to tree‐killing bark beetles and fungi: The ecological stoichiometry of secondary bark beetles, fungi, and a scavenger. Ecological Entomology, 45, 1134–1145. 10.1111/een.12897

[ece310345-bib-0074] Six, D. L. , & Klepzig, K. D. (2021). Context dependency in bark beetle‐fungus symbioses revisited: Assessing potential shifts in interaction outcomes against varied genetic, ecological, and evolutionary backgrounds. Frontiers in Microbiology., 12, 682187. 10.3389/fmicb.2021.682187 34054789 PMC8149605

[ece310345-bib-0075] Six, D. L. , & Livingston, R. L. (2023). Distribution and taxonomic reclassification of the mycangial fungus of the fir engraver, *Scolytus ventralis* . Symbiosis, 89, 123–131.

[ece310345-bib-0076] Six, D. L. , & Paine, T. D. (1996). *Leptographium pyrinum* is a mycangial fungus of *Dendroctonus adjunctus* . Mycologia, 88, 739–744. 10.1080/00275514.1996.12026711

[ece310345-bib-0077] Six, D. L. , & Paine, T. D. (1998). The effects of mycangial fungi on development and emergence of *Dendroctonus ponderosae* and *D. jeffreyi* . Environmental Entomology, 27, 1393–1401. 10.1093/ee/27.6.1393

[ece310345-bib-0078] Thrall, P. H. , Hochberg, M. E. , Burdon, J. J. , & Bever, J. D. (2007). Coevolution of symbiotic mutualists and parasites in a community context. Trends in Ecology and Evolution, 22, 120–126. 10.1016/j.tree.2006.11.007 17137675

[ece310345-bib-0080] Vanderpool, D. , Bracewell, R. R. , & McCutcheon, J. P. (2017). Know your farmer: Ancient origins and multiple independent domestications of ambrosia beetle fungal cultivars. Molecular Ecology, 27, 2077–2094. 10.1111/mec.14394 29087025

[ece310345-bib-0081] Wade, M. J. (2007). The co‐evolutionary genetics of ecological communities. Nature Review Genetics, 8, 185–195. 10.1038/nrg2031 17279094

[ece310345-bib-0083] Weiblen, G. D. , & Treiber, E. L. (2015). Evolutionary origins and diversification of mutualism. In J. L. Bronstein (Ed.), Mutualism (pp. 37–56). Oxford University Press.

